# Chromosome-Scale, Haplotype-Resolved Genome Assembly of Non-Sex-Reversal Females of Swamp Eel Using High-Fidelity Long Reads and Hi-C Data

**DOI:** 10.3389/fgene.2022.903185

**Published:** 2022-05-18

**Authors:** Hai-Feng Tian, Qiaomu Hu, Hong-Yi Lu, Zhong Li

**Affiliations:** ^1^ Yangtze River Fisheries Research Institute, Chinese Academy of Fishery Sciences, Wuhan, China; ^2^ College of Fisheries, Huazhong Agricultural University, Wuhan, China

**Keywords:** *Monopterus albus*, non-sex-reversal female, phased haplotype, genome assembly, HIFI

## Abstract

The Asian swamp eel (*Monopterus albus*) is an excellent model species for studying sex change and chromosome evolution. *M. albus* is also widely reared in East Asia and South-East Asia because of its great nutritional value. The low fecundity of this species (about 300 eggs per fish) greatly hinders fries production and breeding programs. Interestingly, about 3–5% of the eels could remain as females for 3 years and lay more than 3,000 eggs per fish, which are referred to as non-sex-reversal (NSR) females. Here, we presented a new chromosome-level genome assembly of such NSR females using Illumina, HiFi, and Hi-C sequencing technologies. The new assembly (Mal.V2_NSR) is 838.39 Mb in length, and the N50 of the contigs is 49.8 Mb. Compared with the previous assembly obtained using the continuous long-read sequencing technology (Mal.V1_CLR), we found a remarkable increase of continuity in the new assembly Mal.V2_NSR with a 20-times longer contig N50. Chromosomes 2 and 12 were assembled into a single contig, respectively. Meanwhile, two highly contiguous haplotype assemblies were also obtained, with contig N50 being 14.54 and 12.13 Mb, respectively. BUSCO and Merqury analyses indicate completeness and high accuracy of these three assemblies. A comparative genomic analysis revealed substantial structural variations (SVs) between Mal.V2_NSR and Mal.V1_CLR and two phased haplotype assemblies, as well as whole chromosome fusion events when compared with the zig-zag eel. Additionally, our newly obtained assembly provides a genomic view of sex-related genes and a complete landscape of the MHC genes. Therefore, these high-quality genome assemblies would provide great help for future breeding works of the swamp eel, and it is a valuable new reference for genetic and genomic studies of this species.

## Introduction

*Monopterus albus*, commonly known as the Asian swamp eel or rice field eel, is widely distributed throughout Asia, from northern India and Burma to China, Asiatic Russia, Japan, and the Indo-Malayan Archipelago. *M. albus* is an economical fish in China (Yang et al., 2018), Vietnam (Khanh and Ngan, 2010), Thailand (Amornsakun et al., 2018), Philippines (Silva et al., 2020), and Indonesia (Herawati et al., 2018) due to its flavor and nutritional value. One local strain of *M. albus* mainly farmed in Jianghan Plain has been subjected to consecutive selective breeding programs recently due to its preferred body color and superiority in growth rate and fecundity (Yang et al., 2009), and the high-quality genome assembly of the selecting breed had been sequenced recently (Tian et al., 2021).

As a famous hermaphroditic protogynous freshwater fish, every individual of *M. albus* develops first as a functional female and then transforms into a functional male by sex reversal after their first spawning during its life cycle (Liu, 1944; Liem, 1963). The sex-changing phenomenon has attracted significant research interest, and the underlying sex-change mechanisms became a hot topic in past decades, hence making *M. albus* an excellent model for studying sex change (Cheng et al., 2003). Previously, the sex change in *M. albus* was found to be accompanied by changes in steroid levels (Yeung and Chan, 1987) and activity of enzymes involved in steroid pathways (Tang et al., 1975). Recently, some sex-related genes and microRNAs have been characterized, and their expressions seem to be varied among three genders during the sex-change progress, such as *Dmrt* (Sheng et al., 2014), *AMH* (Hu et al., 2015), *Foxl2* (Hu et al., 2014)*, dax1* (Hu et al., 2015), *p450* (Zhang et al., 2008), *G2* (Qu et al., 2015), *Cyp19a1a* (Liu et al., 2009; Zhang et al., 2013), *gonadal soma-derived factor (gsdf)* (Zhu et al., 2016), *Sox9* (Zhou et al., 2003), *Dmrt1* (Huang et al., 2005), *Jnk1* (Xiao et al., 2010), and mal-miR-430a, mal-miR-430b, and mal-miR-430c (Gao et al., 2014; Zhang et al., 2021). Moreover, a batch number of genes with different expression profiles between females and males was characterized through high-throughput RNA-seq (Chi et al., 2017). Lastly, epigenetic modification has been suggested playing important roles in sex reversal, such as the histone deacetylation and methylation of *cyp19a1a* (Zhang et al., 2013) and histone acetylation, methylation, and ubiquitination of *dmrt1* (Lai et al., 2021). No heteromorphic chromosome has been found in *M. albus* (Ma and Shi, 1987). Altogether, the underlying mechanism of sex changes in *M. albus* remains unknown yet (Hilles et al., 2018). The classification of these triggering sex-reversal mechanisms would help understand the diversity and evolution of such an intriguing mechanism and would provide significant help to future breeding programs of this aquaculture species. Furthermore, *M. albus* has the smallest haploid number (2n = 24) of chromosomes among most freshwater fishes (2n = 24–446), and it is also an ideal material for chromosomal evolution studies (Supiwong et al., 2019; Cheng et al., 2020).

Generally, female individuals of *M. albus* obtain well-developed ova during 1–1.5 years of age (∼20 cm long), and intersex individuals have a body length of 30–40 cm (Susatyo et al., 2020; Cheng et al., 2021a). The fecundity of *M. albus* is about 200–300 eggs per fish at about 1 year of age (Susatyo et al., 2020; Yue et al., 2021). Such low fecundity greatly hinders large-scale aquaculture production and breeding works of swamp eel. Generally, the majority of the females of swamp eel complete sex reversal stage in about 8–30 weeks when they are 2 and 3 years old. However, few female individuals remain as functional female when they are over 55 cm and 10 years old (Chan and Phillips, 1967; Yang et al., 2008; Yang and Xiong, 2010). During our breeding work, we also found that few (3–5%) individuals remain as females and could consecutively spawn three more years after their first spawning, and they were referred to as non-sex-reversal (NSR) females. Such NSR females could reach more than 50 cm in total length and lay more than 3,000 eggs per fish (our unpublished observations). They are excellent materials for studying the mechanisms of sex reversal and breeding works. Although a collapsed haplotype assembly genome of *M. albus* (Mal.V1_CLR) has been obtained using the continuous long-read sequencing technology (Tian et al., 2021), improvement in the *de novo* assembly of genomes is still to be desired.

Recent advances in the long-read sequencing technology have allowed a single molecule to be sequenced multiple times to produce long high-fidelity reads (HiFi) with a base level accuracy of 99.9% and hence drastically improve the base quality of the assembly (Wenger et al., 2019) and enable to phase the genome into haplotypes (Porubsky et al., 2021). *Mastacembelus armatus*, a closely related species that belongs to the order Synbranchiformes, also experiences female-to-male sex changes and is proved to possess neo-sex chromosomes, although cytogenetically all chromosome pairs are homomorphic (Xue et al., 2021). Hence, we presented a *de novo* chromosome-scale assembly of NSR females using HiFi reads with the Hifiasm assembly tool. The final assembly Mal.V2_NSR is 838.39 Mb in length with the contig and scaffold N50s of 49.8 and 68.68 Mb, respectively, showing a significant improvement in continuity compared with the assembly Mal.V1_CLR. Meanwhile, two highly contiguous haplotype assemblies were also obtained, with the contig N50 being 14.54 and 12.13 Mb, respectively. The new assembly Mal.V2_NSR is annotated with 22,704 protein-coding genes and contains 97.3% of the core set of conserved actinopterygian orthologs, and there are 331 more predicted genes in it than in the previous assembly Mal.V1_CLR. BUSCO and Merqury analyses indicate completeness and high accuracy of all three assemblies. The comparative genomic analysis revealed substantial structural variations (SVs) between Mal.V2_NSR and Mal.V1_CLR and two phased haplotype assemblies, as well as whole chromosome fusion events when compared with the zig-zag eel. The newly almost complete chromosome-level assemblies would provide resources for the future genomics-assisted breeding program and would be a valuable new reference for genetic and genomic studies of this species.

## Materials and Methods

### Animals and Tissue Sampling

The experimental procedure was approved by the Animal Experimental Ethical Inspection of Laboratory Animal Centre, Yangtze River Fisheries Research Institute, Chinese Academy of Fishery Sciences (ID Number: 2020-THF-01).

Twelve non-sex-reversal female individuals were obtained from a breeding farm in Xiantao City, Hubei province. Their total length is 61.53 (±5.09) cm, and their weight is 289.28 (±93.98) grams (Supplementary Table S1). The fish were immediately dissected after anesthesia with MS-222. The tissue was flash-frozen in liquid nitrogen and stored at −80°C until DNA isolation and sequencing were performed. The muscle tissue of one fish was collected for whole-genome sequencing and Hi-C library construction, and different tissues (the brain, muscle, skin, liver, spleen, heart, stomach, intestine, kidney, and ovary) of another fish were collected and used for transcriptome sequencing.

### Library Preparation and Sequencing

Total genomic DNA was extracted from the muscle using the standard phenol/chloroform method, and the integrity of the genomic DNA molecules was checked by agarose gel electrophoresis. Both the PacBio Sequel II platform and the Illumina NovaSeq platform were applied for genomic sequencing to generate long and short genomic reads, respectively.

For the Illumina NovaSeq platform (San Diego, CA, USA), a pair-end library was constructed with an insert size of 350 base pairs (bp), according to the protocol provided by the manufacturer. Large-insert (15 kb) single-molecule real-time circular consensus sequencing (HiFi CCS) library preparation was conducted, following the Pacific Biosciences recommended protocols as described in our previous work (Tian et al., 2021). The constructed library was sequenced on one SMRT cell on the PacBio RS II machine (Pacific Biosciences of California, Inc.). To obtain a chromosome-level genome, 1 g of muscle tissue from the same individual was used for Hi-C library construction. The Hi-C library was prepared from cross-linked chromatins using a standard Hi-C protocol; then, the library was sequenced using the Illumina NovaSeq 6000 instrument (Illumina), as in our previous study (Tian et al., 2021).

We also performed RNA sequencing to generate transcriptome data on the Illumina NovaSeq platform for gene model prediction. RNA was extracted from nine tissues mentioned previously using the TRIzol reagent (Invitrogen). RNA purity was checked using the kaiaoK5500VR spectrophotometer (Kaiao, Beijing, China), and RNA integrity and concentration were assessed using the RNA Nano 6000 Assay Kit of the Bioanalyzer 2100 system (Agilent Technologies, CA, USA). A total amount of 2 μg RNA per sample was mixed, and sequencing libraries were generated according to the manufacturer’s recommendations and sequenced using the Illumina NovaSeq 6000 platform.

### Genome Assembly and Phasing

The genome size, heterozygosity, and repeat content were first estimated through a k-mer analysis with Jellyfish v2.2.10 (Marcais and Kingsford, 2011). HiFi reads were assembled by Hifiasm, version 0.15 (r327) (https://github.com/chhylp123/hifiasm/, last accessed on 16 July 2021) (Cheng et al., 2021b). gfatools (https://github.com/lh3/gfatools) was used to convert sequence graphs from the GFA to FASTA format. Duplicated haplotigs and artifacts were identified and removed using the purge_haplotigs (https://bitbucket.org/mroachawri/purge_haplotigs, last accessed on 10 October 2021) (Roach et al., 2018). To order and orient contigs into pseudomolecules, Hi-C reads were mapped to the draft assembly with Juicer v1.5.6 (Durand et al., 2016b); then, the candidate chromosome-length assembly was obtained using the 3D-DNA (v201008) (Dudchenko et al., 2017) pipeline to correct misjoins, order, orient, and anchor contigs from the draft assembly. Last, the assembly was manually checked and refined with Juicebox v. 1.11.08 (Durand et al., 2016a) for quality control and interactive correction. This newly obtained genome assembly was named Mal.V2_NSR. Moreover, two sets of haplotype-resolved, phased contig (haplotig) assemblies were generated using hifiasm with a combination of HiFi reads and paired-end Hi-C reads, and they were scaffolded into a chromosome-scale assembly as mentioned previously. These two phased haplotype assemblies were referred to as Hap1 and Hap 2, respectively.

### Assessment of Assembly Quality

First, the HiFi reads and Illumina paired-end reads were mapped to the assembly by Minimap2 and BWA, respectively, to evaluate the completeness (Li, 2018). The mapping rates and the genome coverages were calculated by Samtools (version 0.1.19) (Li et al., 2009). Picard, GATK (version 3.1-1) was used to call SNPs (McKenna et al., 2010). Second, the completeness of the primary assembly was assessed by Benchmarking Universal Single-Copy Orthologs (BUSCO) (v4.0.6) (Simao et al., 2015) using the Actinopterygii_odb9 database as the reference data set. Third, the consensus quality values (QVs) of the obtained three assemblies were also computed by Merqury version 1.3 (https://github.com/marbl/merqury, last accessed on 21 October 2021) (Rhie et al., 2020). Last, to assess the quality of the three assemblies, CHROMEISTER (Perez-Wohlfeil et al., 2019) was used to perform a pairwise comparison between Mal.V2_NSR and Mal.V1_CLR, and Hap1 and Hap2.

### Repeat Characterization and Genome Annotation

Repetitive elements were identified by homolog searching using RepeatMasker and RepeatProteinMask (Chen, 2004) with Repbase (version 23.08) libraries (Jurka et al., 2005; Bao et al., 2015) and identified *de novo* assemblies by using RepeatModeler, Tandem Repeats Finder (TRF) (Benson, 1999), and LTR-FINDER (Xu and Wang, 2007).

Based on the repetitive-element masked genome, genes were predicted using *ab initio* approach, and homolog searching. For *ab initio* gene prediction, AUGUSTUS v3.3.2 (Stanke et al., 2006), GeneScan (Aggarwal and Ramaswamy, 2002), and GlimmerHMM were used for the prediction of genes in the repeat-masked genome. For homology-based predictions, protein sequences of six closely related teleost species, including *Acanthochromis polyacanthus* (GCF_002109545.1), *Amphiprion ocellaris* (GCF_002776465.1), *Anabas testudineus* (GCF_900324465.1), *Astatotilapia calliptera* (GCF_900246225.1), *Mastacembelus armatus* (GCF_900324485.1), and our previously assembled genome of *M. albus* (Mal.V1_CLR) (GWHBEHV00000000) were downloaded from the National Center for Biotechnological Information (NCBI) database and National Genomics Data Center (https://ngdc.cncb.ac.cn/) and were mapped onto the newly assembled *M. albus* genome using BLASTN. The alignment hits were joined by Solar software (Yu et al., 2006). Subsequently, GeneWise v2.2.0 (Birney et al., 2004) with default options was used for homologous annotation. In addition, RNA-seq reads were directly mapped to the assembled genome to identify putative exon regions using the TopHat package (v2.1.1) (Trapnell et al., 2009) and Cufflinks (v2.2.1) (Ghosh and Chan, 2016). Finally, all the gene models were merged, and redundancy was removed by MAKER (Cantarel et al., 2008) and integrating the CEGMA (Parra et al., 2007) results based on the HiCESAP process.

All predicted genes were functionally annotated, as previously described (Tian et al., 2021). Various databases, including NCBI non-redundant protein (nr), SwissProt (Boeckmann et al., 2003), Kyoto Encyclopedia of Genes and Genomes (KEGG) (Ogata et al., 1999), TrEMBL (Boeckmann et al., 2003), InterPro (Zdobnov and Apweiler, 2001), PFAM (Mistry et al., 2021), eukaryotic orthologous groups of proteins (KOG) (Tatusov et al., 2001), TFs, and Gene Ontology (GO) (Ashburner et al., 2000), were used to functionally annotate the predicted protein-coding genes.

For non-coding RNAs, microRNA (miRNA) and small nuclear RNA (snRNA) were predicted using INFERNAL v1.1.2 (Nawrocki and Eddy, 2013) and the Rfam database (release 13.0) (Griffiths-Jones et al., 2003; Kalvari et al., 2018). Transfer RNA (tRNA) and ribosomal RNA (rRNA) were identified using tRNAscan-SE v1.3.1 (Lowe and Eddy, 1997) and RNAmmer (v1.2, http://www.cbs.dtu.dk/services/RNAmmer/), respectively.

### Comparative Genomic Analysis

Synteny and Rearrangement Identifier (SyRI) (https://github.com/schneebergerlab/syri) v1.5 (Goel et al., 2019) was used to identify structural variations (SVs) between the newly obtained assembly Mal.V2_NSR and the previous assembly Mal.V1_CLR and the two phased assemblies Hap 1 and Hap 2. Minimap2 v2.23-r1111 was used to perform the alignment with the following parameter setting “-ax asm5 –eqx,” and then, SVs were detected. The output of SyRI was plotted with the script plotsr. The SVs from SyRI were reclassified as presence-absence variations (PAVs) as follows: the CPL, DEL, DUP/INVDP (loss) variants, and the sequences in NOTAL were converted as Absence SVs, and the CPG, INS, DUP/INVDP (gain) variants, and the query sequences in NOTAL were converted as Presence SVs. Genes affected by these PAVs were identified with the BEDTools (v2.30.0) (Quinlan and Hall, 2010) by comparing their absolute positions to the genome annotations. We performed a Gene Ontology enrichment analysis to find enriched biological functions by using TBtools (Chen et al., 2020).

The pairwise genome comparison between Mal.V2_NSR and the genome of *M. armatus* (fMasArm1.2, GCF_900324485.2) (Rhie et al., 2021) was performed by MCScanX (Python version) (Tang et al., 2008; Wang et al., 2012). Syntenic regions were identified with the LAST results, and dot plots for genome pairwise synteny visualization were generated using the command “python -m jcvi.graphics.dotplot.” The synteny pattern of the two compared genomes was generated using the command “python -m jcvi.compara.synteny depth -histogram.”

### Sex-Related Genes and Major Histocompatibility Complex Genes

Previous studies have revealed multiple sex-related genes that might be involved in sex change, gonadal differentiation in *M. albus*, and other teleosts (Zhang et al., 2008; Wu et al., 2012; Hu et al., 2014; Hu et al., 2015; Gao et al., 2016; Cai et al., 2017; Yi et al., 2019; Ding et al., 2020; Chen et al., 2022; Feng et al., 2022). To investigate a genomic survey of these genes in the assembled genome, these genes were directly searched in the annotated genome using gene name or identified with tBlastn (v2.9.0+) (Camacho et al., 2009) in the annotated genome. Major histocompatibility complex (MHC) genes play important roles in the immune response of vertebrates and exhibit high degrees of genetic diversity, and their genome organization varies greatly among fish (Hu et al., 2022). To obtain an overview of the genome organization of MHC genes in swamp eels, MHC genes were searched by their gene names and characterized by domain analysis and then classified as class I and II by searching against a non-redundant (nr) protein database with BlastP (last accessed on 10 March 2022).

## Results

### Genome Assembly and Assessment

**FIGURE 1:**
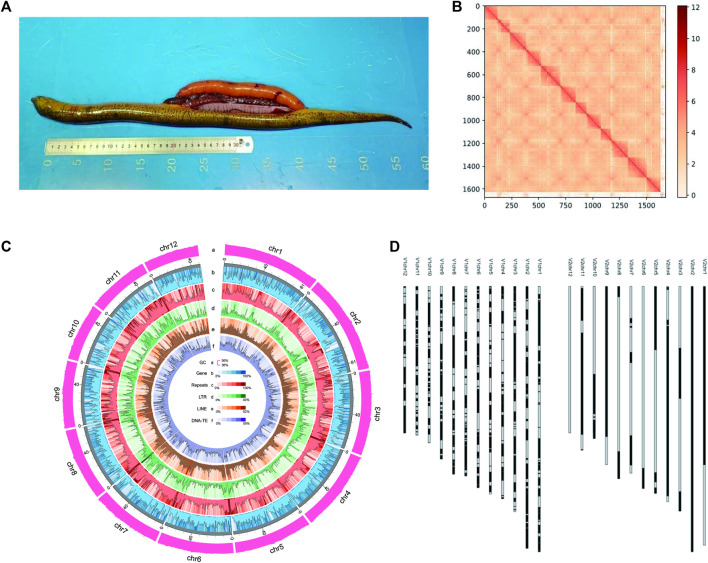
Genome assembly of non-sex-reversal (NSR) female of *M. albus*. **(A)** Picture of an NSR swamp eel. **(B)** Heatmap of the *M. albus* Hi-C assembly. Red blocks refer to the intensity of interaction among sequences. **(C)** Genome features of the primary assembly (Mal.V2_NSR). From a to f: a, GC content; b, Gene density; c, Repeats; d, LTR; e, LINE, and f, DNA transposable elements (TEs) and RNA transposable elements (RNA-TEs). **(D)** Visualization of improvements in assembly contiguity. Ideogram of Mal.V1_CLR (left) and Mal.V2_NSR (right) assemblies.

A total of 89.84-gigabyte (Gb) Illumina clean reads were obtained by using Illumina paired-end libraries (Supplementary Table S2), and the genome size was estimated to be 830 Mb based on 17-nt *k*-mer length with a mean *k*-mer coverage depth of 71-fold (Supplementary Figure S1). A total of 1,764,134 HiFi reads consisting of 29,369,894,529 bp were obtained (Supplementary Table S2) on the PacBio Sequel II platform, with the average lengths of 16,648.34 bp and N50 16,790 bp. Using 35.4× high-fidelity (HiFi) reads, an 843-Mb primary assembly was obtained with Hifiasm, including 384 contigs. The contig N50 of the primary assembly was 45.38 Mb. Then, a total of 116.59 Gb clean data obtained from the Hi-C library (Supplementary Table S2) were used to scaffold the contigs to a chromosome-scale assembly. According to the aforementioned mapping strategy, the proportion of valid interaction pairs was 87.6%, indicating the high quality of the Hi-C library. As a result, 384 contigs were successfully anchored and oriented into 12 chromosomes (Figure 1B). More than 98.27% of contigs longer than 100 kb were anchored on chromosomes, exhibiting an excellent anchoring rate for the chromosome assembly. The final assembly was 838.39 Mb (Figure 1C), with a contig N50 of 49.8 Mb and scaffold N50 of 68.68 Mb, and included 38.66 Mb additional sequences (Table 1). This new assembly was named Mal.V2_NSR, and the 12 chromosomes were numbered based on similarity to the Mal.V1_CLR assembly (Accession No. GWHBEHV00000000). The Mal.V2_NSR assembly was highly concordant with Mal.V1_CLR, according to the synteny plot (Supplementary Figure S2), and it is more contiguous than the Mal.V1_CLR (Figure 1D), with a 20- fold increase in contig N50 values (Table 1). Noticeably, chromosomes 2 and 12 were assembled into a single contig (Figure 1D; Table 1). Meanwhile, with the help of the hifiasm assembler, two sets of haplotigs (phased haplotype-resolved contigs) were obtained and scaffolded into pseudochromosomes with Hi-C data. The obtained two phased haplotype assemblies (referred to as Hap1 and Hap2) are ultra-continuous, with contig N50s of 14.53 Mb for Hap1 and 12.13 Mb for Hap2, respectively (Table 1). The quantitative statistics for these three assemblies are listed in Table 1.

**TABLE 1:** Comparisons of the genomic features for the Mal.V2_NSR, Hap1, Hap2, and Mal.V1_CLR assemblies.

Feature	Mal.V1_CLR	Mal.V2_NSR	Hap1	Hap2
Genome size	799,723,043	838,386,204	823,638,986	824,747,869
GC content	41.4	41.5	41.5	41.4
Contig N50 (bp)	2,478,495	45,384,804	14,535,311	12,130,162
Scaffold N50 (Mb)	67.24	68.68	67.85	67.76
chr1	87968752	87968752	86759400	88254400
chr2	87516135	90197515	90917400	90820609
chr3	75258030	76372968	75537254	75998379
chr4	70524527	73151148	72292532	72524261
chr5	69375753	70326400	70800232	69783651
chr6	67051601	68680745	67849023	67756700
chr7	63368872	63480983	65243340	61584783
chr8	62765706	65388200	64740500	65087849
chr9	57703348	60645400	58130864	60541200
chr10	52267621	51709500	53479816	51461713
chr11	49704940	55667652	51005900	53312973
chr12	49006820	49804000	50305268	50059400
QV	41.35	52.448	52.2337	53.0244

The mapping rate of CCS reads was 99.98% (covering 99.97% of the total length of the genome, among which, 99.78% with a coverage depth ≥4×, 99.18% with a coverage depth ≥10×, and 92.02% with a coverage depth ≥20×) (Supplementary Table S3). The mapping rate of the whole Illumina short reads by BWA was 99.8% (Supplementary Table S3), which means that almost all sequencing data were represented (covering 99.96% of the total genome length, among which, 99.91% with a coverage depth ≥4×, 99.82% with a coverage depth ≥10×, and 99.65% with a coverage depth ≥20×, showing high coverage). Both coverage rates of PacBio sequencing and Illumina sequencing were consistent and relatively high. The homozygous rate of SNPs and indels were both as low as 0%, suggesting the accuracy of the genome assembly was very high; the heterozygous rate of SNPs and indels were as low as 0.144% and 0.027%, respectively, indicating that genome heterozygosity was low. The BUSCO analysis showed that the primary assembly covered 97.3% of complete BUSCOs (C) and 94.4% of universal single-copy genes, and the Hap 1 and Hap 2 assemblies covered 97.1% and 97.3 of complete BUSCOs, respectively (Supplementary Table S4, and Supplementary Figure S3). The whole-genome comparison between Mal.V2_NSR and Mal.V1_CLR and two phased haplotype assemblies (Hap 1 and Hap2) indicated the distance scores are very low, and very significant signals are found on the diagonals (Supplementary Figure S2), indicating the good quality of our assemblies. Additionally, the consensus quality values (QVs), which represent the Phred-scaled probability of the base error in the assembly, were 52.48 for Mal.V2_NSR, 52.23 for Hap1, and 53.02 for Hap2, indicating a relatively high assembly accuracy for the newly obtained V2-NSR and two phased assemblies (Table 1).

### Genome Annotation

A complete summary of the repeat element composition identified in the Mal.V2_NSR assembly is available in Supplementary Table S5. DNA transposons (21.01%), long interspersed nuclear elements (LINEs, 18.07%), and long terminal repeats (LTRs, 10.32%) are the top three categories of repetitive elements in this assembly (Supplementary Figure S4). Altogether, a total of 47.99% of the genome comprised repeats, which is slightly higher than our previously published Mal.V1_CLR assembly. After the analysis, 273 miRNAs, 42,869 tRNAs, 3,933 rRNAs, and 964 snRNAs were annotated in the Mal.V2_NSR assembly (Supplementary Table S6).

Finally, a total of 22,704 protein-coding genes were identified (Supplementary Table S7), of which 19,516, 21,813, 22,066, 20,356, and 15,457 protein-coding genes were annotated in the SwissProt, KEGG, TrEMBL, InterPro, and GO databases, respectively (Supplementary Table S8). BUSCO was also used to test the completeness of the genome annotation with the actinopterygii_odb9 database, which showed that 93.9% complete and 1.0% fragmented conserved single-copy orthologs were predicted for the Mal.V2_NSR assembly.

### Comparative Genomic Analysis

**FIGURE 2:**
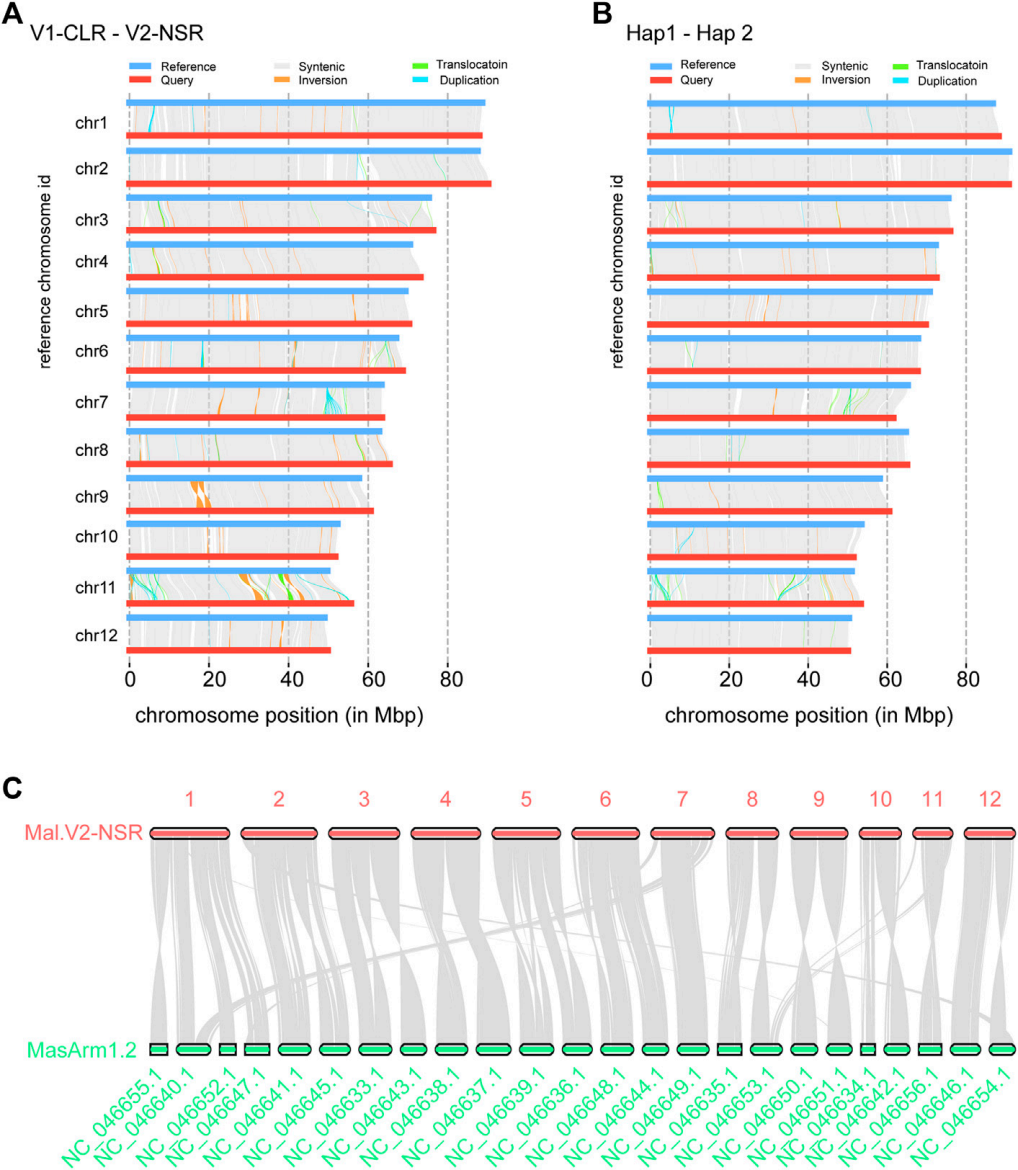
Genome comparative analysis. Synteny and rearrangement (SyRI) plot of chromosomes of the Mal.V2_NSR assembly (Query) against Mal.V1_CLR assembly (Ref) **(A)**, and Hap1 (Query) against Hap2 (Ref) **(B)**. **(C)** Chromosome-conserved synteny between Mal.V2_NSR and *M. armatus* (MasArm 1.2). Syntenic blocks are linked by shaded bands, and non-syntenic (non-matching) regions are shown as white gaps between Ref and Query.

Comparing the new assembly (Mal.V2_NSR) with the reference Mal.V1_CLR assembly using the whole-genome comparison tool SyRI, we found 703–707 Mb of collinear regions in each of these two assemblies (Figure 2A). In addition, the SyRI analysis identified 268 inversions (∼15 Mb), 540 translocations (∼5 Mb), and 137 duplications in Mal.V1_CLR (1.5 Mb) and 890 duplications in Mal.V2_NSR (4.38 Mb), and we also identified that 78.73 Mb of Mal.V1_CLR and 88.50 Mb of Mal.V2_NSR DNA sequences were not aligned (Supplementary Table S9). The large PAVs (>1,000 bp) contain 2,474 genes in Mal.V1_CLR and 2,616 genes in Mal.V2_NSRv. Gene ontology enrichment analysis for these PAV genes identified 19 GO terms including six biological processes, nine molecular functions and four cellular components that were significantly enriched (Supplementary Figure S5). Meanwhile, comparing the two phased haplotype assemblies allowed us to gain insights into the haplotype diversity within NSR female individuals, including 224 inversions (∼3.8 Mb), 276 translocations (∼1.75 Mb), and 25 duplications (∼0.22 Mb) in Hap1, and inversions (∼3.8 Mb), 276 translocations (∼1.75 Mb), and 272 duplications (∼1.74 Mb) in Hap2, making up about 8% of the assembled sequence (66.04 and 69.64 Mb) in each of the haplotype assemblies (Figure 2B, Supplementary Table S10).

Moreover, the synteny analysis for the Mal.V2_NSR assembly and *M. armatus* v1.2 (GCF_900324485.2) using homolog gene pairs was performed (Figure 2C). As a result, a total of 176 collinear blocks and 16,553 gene pairs were identified between swamp eel and *M. armatus*. Noticeably, nine chromosomes (chromosomes 2, 3, 4, 5, 6, 8, 9, 10, and 12) of *M. albus* harbored one-to-two synteny to the chromosomes of *M. armatus*, and chromosome 1 of *M. albus* showed conserved synteny with four chromosomes of *M. armatus*.

### Sex-Related and Major Histocompatibility Complex Genes

**FIGURE 3:**
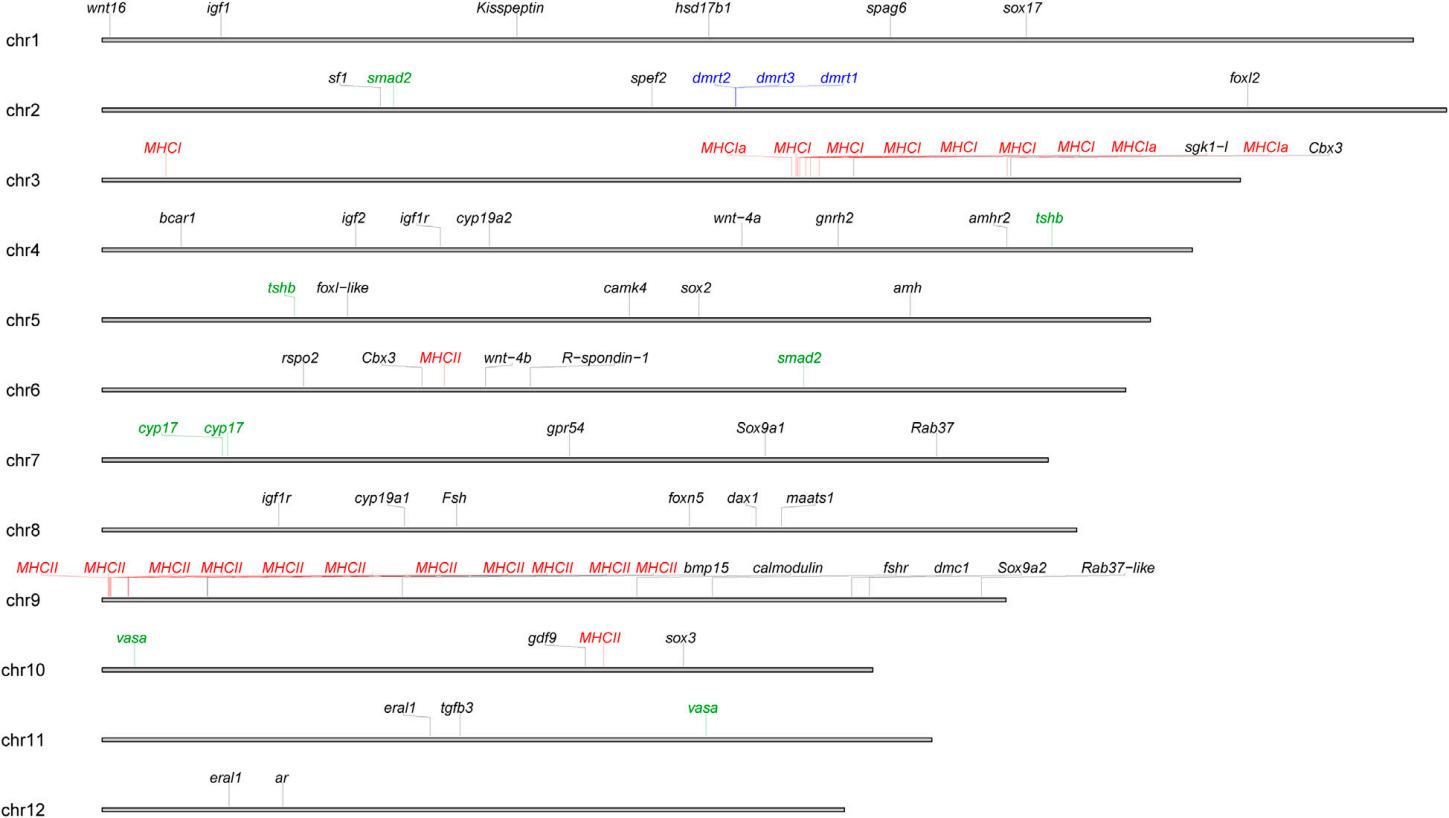
Distributions of sex-related genes and *MHC* genes on the newly obtained assembly (Mal.V2_NSR). The conserved *dmrt1*-*dmrt3*-*dmrt2* gene cluster is shown in blue, *MHC* genes are shown in red, and sex genes with two copies are shown in green.

A total of 59 sex-related genes were found and confirmed by using Blast against the nr database, and they were mapped onto 12 chromosomes (Supplementary Table S11) (Figure 3). Noticeably, a conserved *dmrt1*–*dmrt3*–*dmrt2 (2a)* gene cluster was found on chromosome 2. In addition, two copies of four sex-related genes, *Tsh* (Feng et al., 2022), *Vasa* (Ye et al., 2007), *Smad2* (He et al., 2020), and *cyp17* (Yu et al., 2003)were characterized with only one coding gene previously, and they were mapped to different chromosome positions. Twenty-three MHC genes were identified and classified as class I and class II, respectively (Supplementary Table S12), and they were mapped onto chromosomes 3, 6, 9, and 10 (Figure 3).

## Discussion

As a widely distributed species throughout Asia, *M. albus* is widely cultured in China and other countries of Asia as aquaculture fish because of its economic value. *M. albus* undergoes sequential sex change from female to male after its first spawning at about 1.5 years old. The average fecundity of one-year-old females is only about 300 eggs per fish, and such low fecundity greatly hindered the production of fries and the breeding program, leading to the shortage of functional females in production. Few 10-years-old female individuals of swamp eel found previously (Yang et al., 2008) suggest some individuals of swamp eel could maintain its gender during whole life history. About 3–5% females could remain their gender 3 years or more and lay more than 3,000 eggs per fish in production. These females are referred to as non-sex reversal (NSR) females. According to the relationships between total length with age and von Bertalanffy growth curve (Yang and Xiong, 2010), these NSR female might be over 6 years old. Hence, such NSR female individuals of swamp eel would be valuable resources for studying mechanisms of sex change.

In the present study, we have combined the benefit of highly accurate reads with an improved assembly tool Hifiasm (Cheng et al., 2021b) and Hi-C sequencing technologies, a *de novo* assembly (Mal.V2_NSR), and two haplotype-resolved assemblies (Hap1 and Hap2) of one natural NSR female of *M. albus* were obtained. The higher contig N50 values indicate that all these three assemblies were significantly more continuous than the Mal.V1_CLR assembly. All these analyses indicate that these assemblies were significantly more continuous than the Mal.V1_CLR assembly. The slightly higher BUSCO percentages and QV values indicate the completeness and accuracy of the three assemblies, and the conserved similarity detected using CHROMEISTER further confirmed the quality of these three assemblies.

Genomic structural variation are important contributor to genetic diversity, and have been found facilitating adaptation, shaping complex trait, and even involved in speciation (Feulner and De-Kayne, 2017; Chakraborty et al., 2019; Hamala et al., 2021). Excepting massive syntenic blocks detected by Syri, we found 268 inversions (∼15 Mb), 540 translocations (∼5 Mb), and 137 duplications in Mal.V1_CLR (1.5 Mb) and 890 duplications in Mal.V2_NSR (4.38 Mb). We also identified 78.73 Mb of Mal.V1_CLR and 88.50 Mb of Mal.V2_NSR DNA sequences that were not aligned (Supplementary Table S9). Meanwhile, comparing the two haplotype assemblies allowed us to gain insights into the haplotype diversity within NSR female individuals, including 224 inversions, 276 translocations, and 25 duplications in Hap1 and 272 duplications in Hap2, making up about 8% of the assembled sequence (66.04 and 69.64 Mb) in each of the haplotype assemblies. Structural variation refers to genomic alterations with a wide size range, including inversions, translocations, and duplications (or deletions). A total of 19 GO terms were enriched for the PAV genes specifically for the Mal.V1_CLR and Mal.V2_NSR assembly. Interestingly, threonine-type peptidase activity and threonine-type endopeptidase activity, which are enriched molecular functions for bovine seminal plasma proteins (Gomes et al., 2020), are also enriched in the Mal.V2_NSR assembly, suggesting the genes involved in semen development might be affected by these PAVs in NSR females. Besides, the uncovered heterozygosity between two haplotype assemblies would also be valuable resources to uncovering potential sex-determination regions and/or allele-specific expression that might lead to large phenotypic variations (Zhang et al., 2020).

*M. albus* has 12 acrocentric chromosome pairs (Ji et al., 2003), the lowest chromosome number among teleost fish. Whole-chromosome fusions have been found in this lineage after being separated from medaka (Cheng et al., 2020). Through a comparison with the annotated genome of *M. armatus*, a total of 176 collinear blocks and 16,553 gene pairs were identified between swamp eel and *M. armatus*, and multiple 2:1 chromosome level syntenic alignments between *M. armatus* and *M. albus* were found (Figure 2B). Considering the chromosome number of other species in Synbranchids, for example, *M. cuchia* (2n = 42) (Rishi and Haobam, 1984), *Synbranchus marmoratus* (2n = 42–46) (Carvalho et al., 2012; Utsunomia et al., 2014), *Ophisternon aenigmaticum* (2n = 46) (Nirchio et al., 2011), and *O. bengalense* (2n = 46) (Carvalho et al., 2012) and a reduction in the chromosome number from 2n = 24 to 2n = 18 found in one Asian swamp population native to central Thailand (Supiwong et al., 2019), the chromosome-wide fusion might have occurred very recently and frequently after the divergence of *M. albus* from other species of Synbranchids during evolution and led to the great reduction in the chromosome number of this species.

Fish sex can be very plastic, with the combination of genetic and environmental influences, and the sex determination mechanism shows a high degree of plasticity and complexity correspondingly (Ribas et al., 2017). As a protogynous hermaphroditic fish, no heteromorphic sex chromosome has been found in *M. albus* yet. Batch sex-related genes have been characterized in *M. albus* previously and/or other teleosts and seem to be equally distributed on 12 chromosomes. The *dmrt1*-*dmrt3*-*dmrt2* gene cluster conserved among teleosts (Dong et al., 2020) was found and mapped on chromosome 2, which confirmed a previous postulation based on the Southern blot analysis (Sheng et al., 2014). *M. armatus*, a closely related species of *M. albus* belonging to Synbranchiformes, which is also a sequentially hermaphroditic fish, and a young sex chromosome pair and an almost identical sex-linked region (SLR) between X and Y chromosomes were identified recently (Xue et al., 2021). Moreover, protogynous hermaphrodites have also been widely found in Synbranchidae, such as *Synbranchus marmoratus*, *S. bengalensis*, and *Typhlosynbranchus boueti* (Liem, 1968; Barros et al., 2012; Barros et al., 2017; Mazzoni et al., 2018). A closer inspection of the underlying sex-reversal mechanisms in Synbranchiformes would help understand the diversity and evolution of sex-determining modes.

The major histocompatibility complex (MHC) genes play key roles in antigen recognition and presentation in adaptive immunity, which are believed to have evolved in response to pathogens. The genomic organization of MHC genes varies greatly in teleosts (Hu et al., 2022). Because of the high degree of gene duplication and diversification of MHC genes, only two MHC genes have been characterized (Li et al., 2014; Pan et al., 2018). The high-quality chromosome-level genome assembly provide a new opportunity to address this problem. Here, a total of 23 MHC genes were characterized in the high continuous assembly, and they were mainly mapped on chromosomes 3, 6, 9, and 10 separately. This distribution pattern is similar to the pattern of other teleosts, such as *D. rerio* and rare minnow (*Gobiocypris rarus*) (Dijkstra et al., 2013; Hu et al., 2022). These results would help carry out future studies on the functional diversity of MHC genes.

## Conclusion

In this study, we assembled a chromosome-scale *de novo* genome assembly and two haplotype-resolved genome assemblies of one natural non–sex reversal female of *M.albus* based on Illumina, HiFi, and Hi-C sequencing data. Both the sequence continuity (contig N50) and genome quality (base accuracy and completeness) were higher than those of the previously released swamp eel genome, implicating the advantages of HiFi reads on the *de novo* genome assembly. A comparative genome analysis revealed amounts of SVs between the NSR female and previous assembly, two haplotype assemblies, and the recent massive chromosome fusion in *M. albus*. This chromosome-scale unphased and two phased haplotype genome assemblies will help the characterization of phenotypic- and haplotype-specific mutations and their functions. Furthermore, we provided a genomic survey on the 59 genes potentially associated with the sex change and 23 MHC genes. Altogether, these data provide valuable resources for further biological studies and molecular breeding of this species to obtain the genetic improvement of this economically important teleost fish.

## Data Availability

The raw sequence data reported in this paper have been deposited in the Genome Sequence Archive (Wang et al., 2017) in the National Genomics Data Center (National Genomics Data Center and Partners, 2020), Beijing Institute of Genomics (China National Center for Bioinformation), Chinese Academy of Sciences, under the accession number CRA006424 that are publicly accessible at https://ngdc.cncb.ac.cn/gsa/. Datasets generated during this study have been deposited in the Genome Warehouse in National Genomics Data Center (https://ngdc.cncb.ac.cn/gwh/), under GWHBHOS00000000, GWHBHOR00000000, and GWHBHOQ00000000.
