# Supplementary figures and images for: Chromosome-Scale, Haplotype-Resolved Genome Assembly of Non-Sex-Reversal Females of Swamp Eel Using High-Fidelity Long Reads and Hi-C Data
Source: Front Genet. 2022 May 18;13:903185. doi: 10.3389/fgene.2022.903185 (PMC9165713; doi:10.3389/fgene.2022.903185)

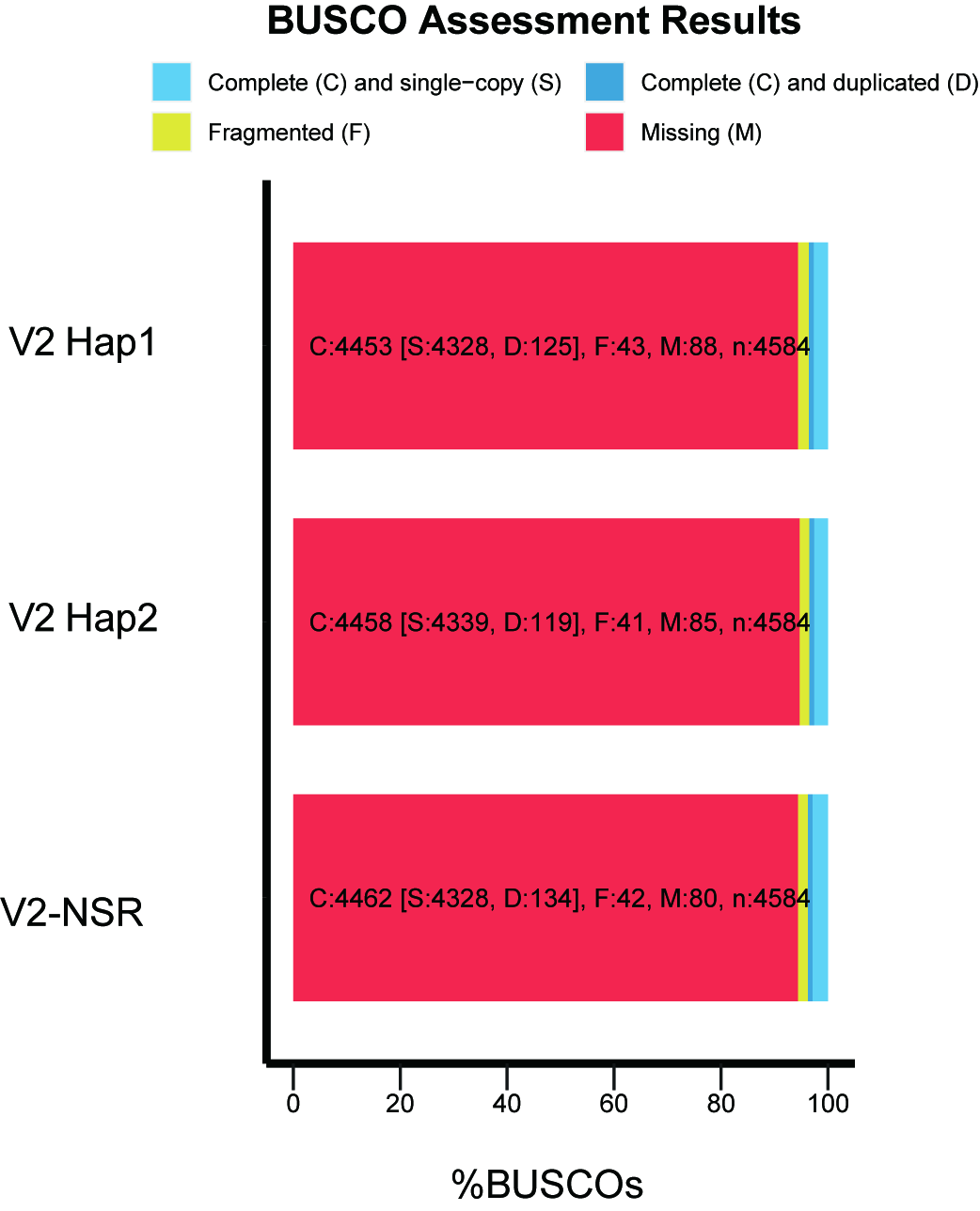

Supplement: Supplementary file 6 [file Image3.tif]

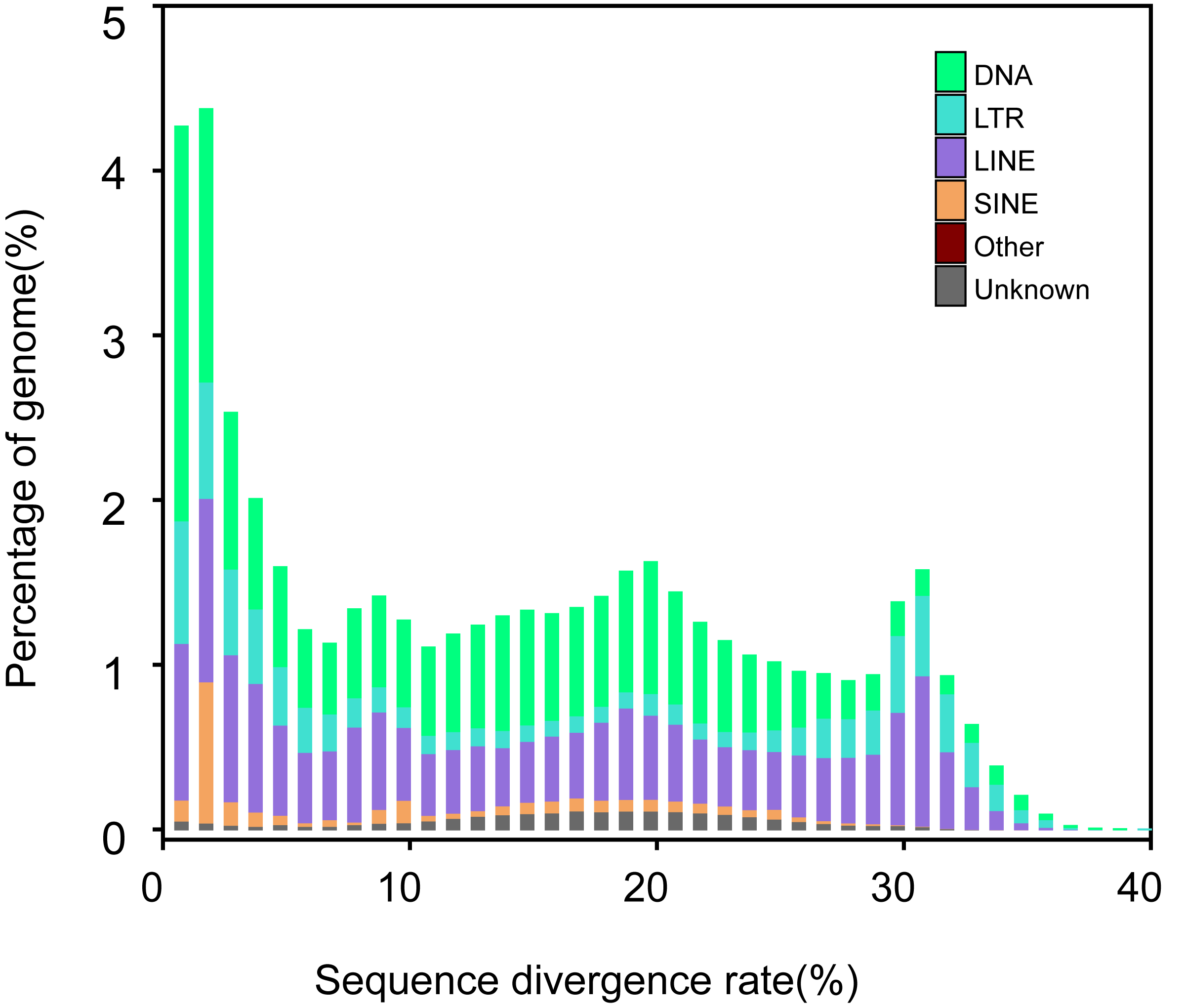

Supplement: Supplementary file 7 [file Image4.tif]

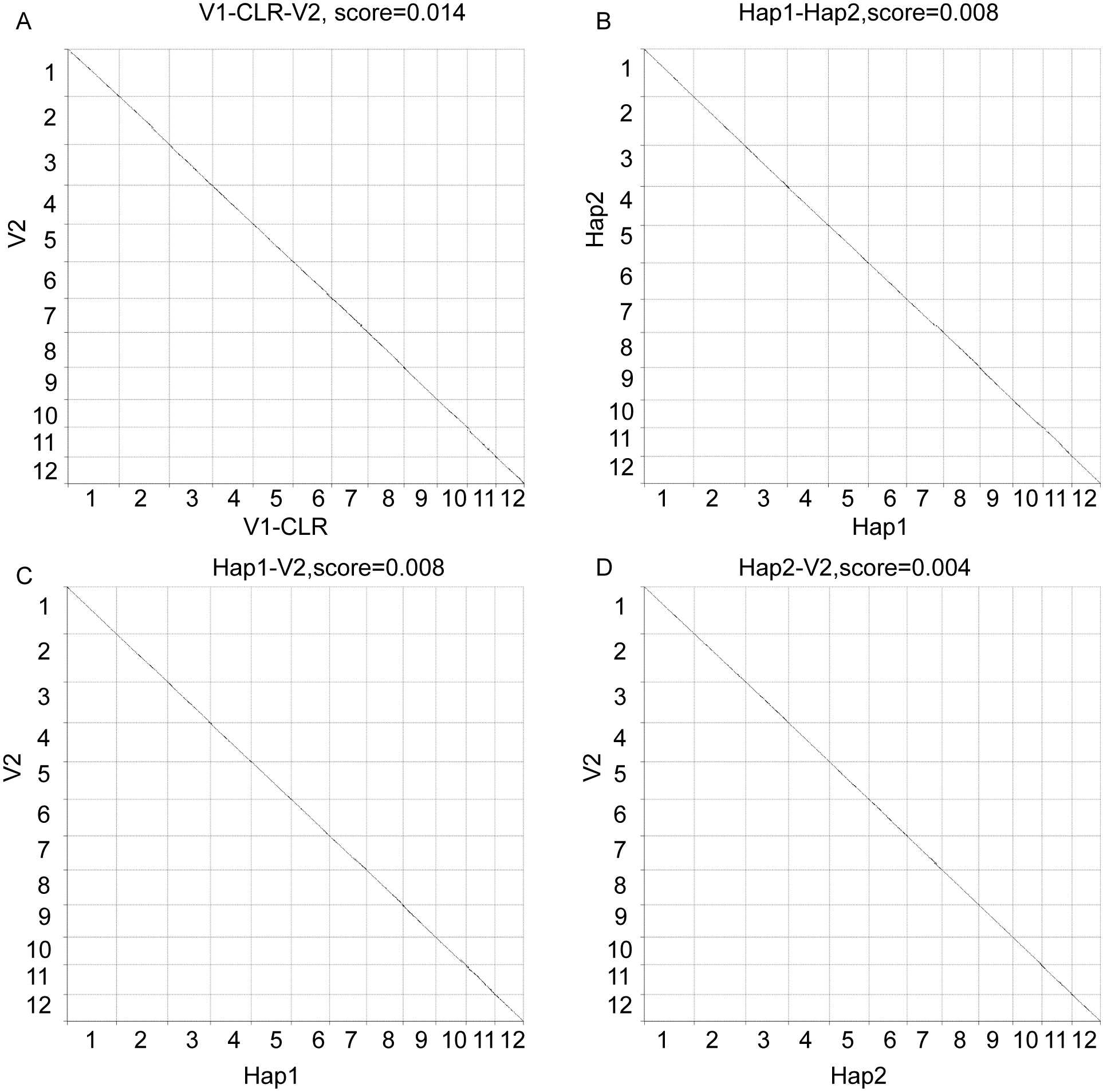

Supplement: Supplementary file 9 [file Image2.tif]

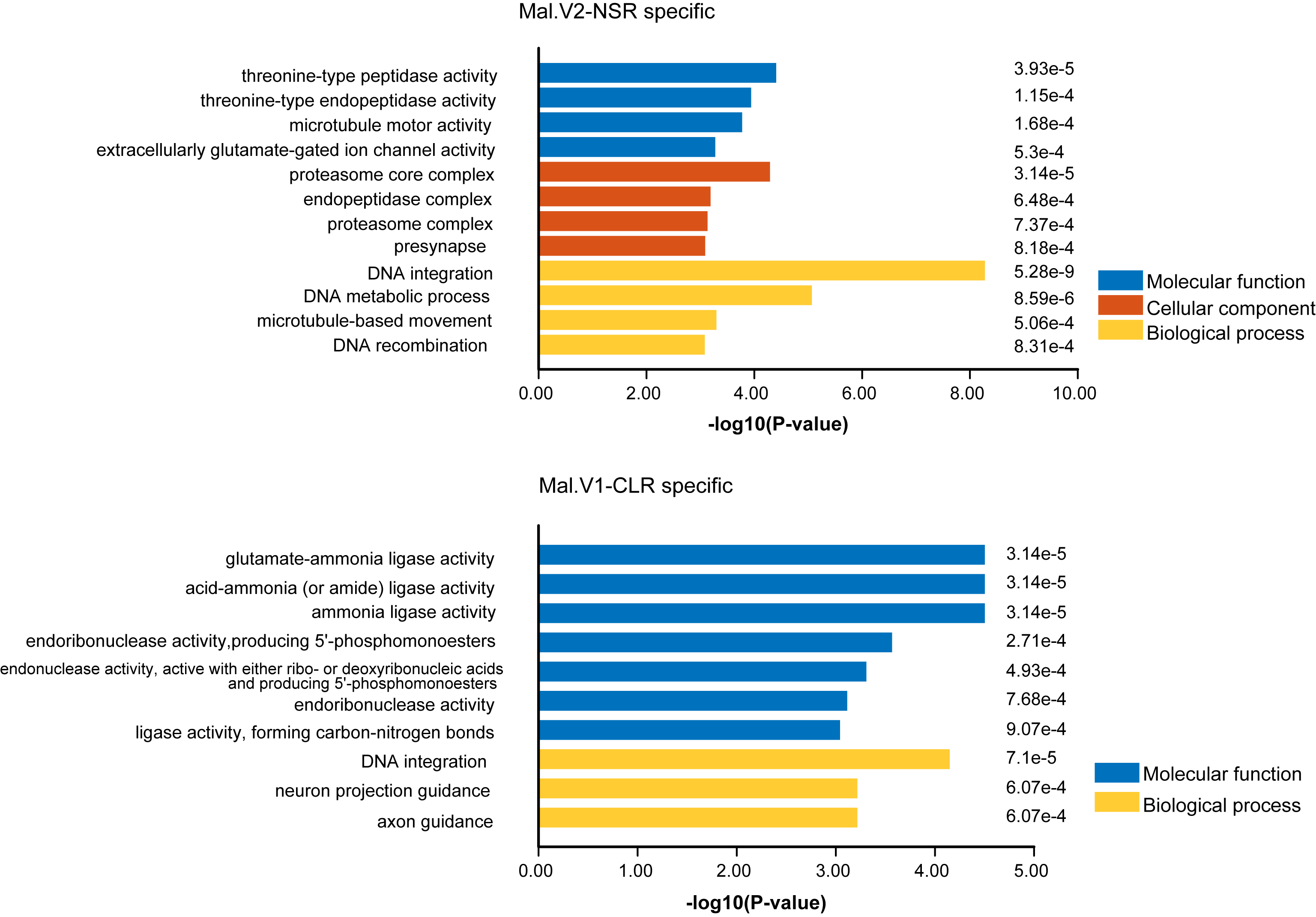

Supplement: Supplementary file 16 [file Image5.tif]
